# Impact of a textile layer on joint optical data and power transfer to in-body devices: a study on an ex vivo approach

**DOI:** 10.1038/s41598-026-48739-1

**Published:** 2026-05-05

**Authors:** Syifaul Fuada, Marcos Katz

**Affiliations:** 1 Centre for Wireless Communications, Faculty of Information Technology and Electrical Engineering, University of Oulu, Oulu, Finland; 2 Oulu’s Infotech, Oulu, Finland

**Keywords:** Forward telemetry, Optical data and power transfer, In-body electronic devices, Clothes, Biological tissue, Energy science and technology, Engineering, Optics and photonics

## Abstract

Wireless connectivity is required in modern in-body electronic devices (IEDs), such as in-body sensors. However, their frequent wireless communication operations will consume more energy than those of conventional IEDs. In addition, energy-limited IEDs require concurrent data and power transfer to maintain uninterrupted operation, enabling medical data communication while continuously supplying energy for battery recharging In this paper, we present a joint optical wireless data and power transfer system for IEDs based on single-carrier transmission, focusing on realistic operating scenarios, with particular emphasis on forward telemetry and energy-harvesting performance. . Commercially available components were employed, including a single-beam 850 nm NIR LED that simultaneously delivers modulated data and optical energy through biological tissue (i.e., ex vivo porcine tissue samples, a representative biological model for human soft-tissue optical propagation). A photodetector receives forward telemetry signals, and a photovoltaic (PV) cell harvests residual optical power concurrently to charge a supercapacitor via a power management integrated circuit (PMIC). In this study, we also account for a real-life factor, namely the impact of clothing on optical light transmission, which may attenuate the incident light depending on fabric type and thickness. Two textile samples were used in this study, representing a thin fabric with high porosity and a thicker fabric with lower porosity (dense yarn). Energy can be harvested through biological tissue during active optical data transmission, thereby providing supplementary energy support for battery-limited IEDs. However, the presence of cloth led to a noticeable decrease in the harvested energy, even with a thin layer. The slower supercapacitor charging is attributed to attenuation of incident optical power by clothing, thereby reducing the PV cell’s output voltage. The study is important for developing future wearable-to-implant links for non-invasive medical applications that account for the presence of clothing.

**Supplementary Information:**

The online version contains supplementary material available at 10.1038/s41598-026-48739-1.

## Introduction

### Wireless communication method for in-body electronic devices (IEDs)

The advent of in-body electronic devices (IEDs) nowadays has transformed patient care, enabling a range of medical functions, including continuous monitoring, diagnosis, and targeted therapy. Depending on the method of placement within the human body, IEDs can be classified into three categories: implanted, ingested, and injected medical devices^1^. Typically, such IEDs are equipped with a wireless communication module to enable bidirectional data exchange with the outside world (i.e., external devices). This includes transmission from an IED to an external device, known as backward telemetry, and from an external device to an IED^2^, referred to as forward telemetry.

Biological tissue is a complex medium that attenuates and scatters wireless signals, complicating the establishment of a reliable wireless link^3^. Several approaches have been explored, including radio frequency (RF), inductive coupling, capacitive body channel communication, optical communication, and ultrasonic transmission^4^. Among these, RF communication is widely used across various domains^5^, including neural monitoring and cardio-respiratory function. In actual implementations, concerns arise about interference when multiple medical devices operating on the same spectrum are simultaneously active for transmission or reception^4^. On the other hand, there are also several concerns in RF communication, such as specific absorption rate (SAR) constraints for human exposure and security or privacy issues, since RF signals are vulnerable to interception or attack. Optical wireless communication system (OWC) presents a compelling alternative to traditional RF-based telemetry due to it offers unique advantages, making it suitable for next-generation IEDs, i.e., (1) electromagnetic interference (EMI)-free, (2) access to large bandwidths to enable high-speed data transmission, (3) no requirement for antennas, and (4) potential for system miniaturization^5^, and (5) provide a safe, private, and secure wireless link^6^. OWC utilizes light as a transmission medium, which inherently exhibits immunity to EMI^7^. This is especially critical in dense clinical environments with wireless electronic devices^4,8^. On the other hand, high-speed wireless data transmission is particularly essential for next-generation IEDs, such as smart pills that transmit high-definition video in real time^9^ and brain implants^9^. Specifically, in^9^, the proposed brain implants feature communication based on OWC combined with sensing capabilities within a shared optical framework (integrated optical link); the system employs a modulated retroreflector (MRR) and diffuse optical tomography (DOT). MRR was used to reduce IED-side energy consumption for communication, while DOT was used for real-time tissue monitoring and detection of biological responses such as inflammation.

Theoretically, optical bands offer much broader bandwidths compared to traditional RF bands, enabling OWCs to exploit unregulated spectrum^8^. Therefore, in-body communication is possible to achieve high data rates by leveraging the extensive bandwidth available in the optical spectrum. A prior study has explored the transmission of modulated light through biological tissue, achieving data rates of up to 40 Mbps^10^. More recent investigations have reported data rates up to 250 Mbps^10^ and 300 Mbps^11^ across several millimeters of porcine skin. OWCs eliminate the need for bulky, large, or metallic antennas, and often complex ones, thereby enabling reduced device footprint, improved biocompatibility, and simplified design considerations^5^. The optical components used for the in-body communication system facilitate greater system miniaturization and energy-efficient circuits^11,12^, particularly through recent advancements in material engineering^13^, and Complementary Metal-Oxide-Semiconductor (CMOS) fabrication^14,15^. Thanks to these technological advances, sub-millimeter-scale devices can also be promoted, which can then be integrated into smaller IEDs with minimal compromise in performance^16^. It can overcome the size constraints of batteries and RF antennas in IEDs^17^. A most recent study showed that a simple bidirectional optical communication between an IED and a wearable skin-surface device is feasible using a soft, flexible, and miniaturizable transceiver platform^18^. Their system used submillimeter-scale ultrathin LEDs (λ = 670 nm) and a photodetector on flexible substrates. A real-time, simple data exchange (characters) and simple medical monitoring (ECG and temperature data) through in vivo skin (~ 3 mm thick) were successfully demonstrated. As IED advances from experimental stages to clinical applications, data security becomes increasingly important^2,12,19^. Physical security is typically preferred due to its simplicity and low resource demands^12^. While data encryption can improve security, it often consumes more power^12,20^. Conversely, OWC is inherently confined to a small area around the body, which significantly reduces the risk of hacking attempts^4^.

### Wireless power transmission (WPT) technologies for in-body electronic devices (IEDs)

Another issue is that IEDs have limited power sources. Batteries remain a significant challenge for their long-term functionality, despite considerable advancements in energy storage technologies. The finite energy capacity constraints often necessitate periodic surgical replacement when the battery nears depletion. These procedures not only increase tissue safety concerns but also contribute to substantial surgical costs from the patient’s point of view^21^. This elevated energy demand poses a critical challenge, particularly for long-term implantation, where minimizing the need for battery replacement is essential. In this context, wireless power transfer (WPT) has emerged as a promising alternative, attracting growing interest in the field of biomedical engineering^22^.

In particular, OWPT has attracted significant research interest and has shown promise for charging IEDs. For example, study in^23^ reported NIR-II subcutaneous photoelectric conversion with skin thermal management validated in vivo, achieving an output power exceeding 500 mW and a photoelectric conversion efficiency of 9.4%. In addition, another study by the same research group demonstrated a photo-thermal-electric converter delivering up to 195 mW under excised tissue and appoximately 20 mW in vivo through 8.5 mm-thick rabbit skin^24^. Later, a study in^25^ showed that an NIR-powered flexible PV cell can support implantable optogenetic stimulation in vivo. Further, a study in^26^ reported up to 9.05 mW/cm² from subdermal solar cell arrays under human skin samples. In addition, a study in^27^ demonstrated an active photonic WPT system using a skin-attachable source patch and an implanted PV array, highlighting the feasibility of wearable-to-IED optical powering through live tissue. More recently, a research group has also investigated several wavelengths (470, 530, 730, 810, and 850 nm) to power IED, where the highest efficiency in power delivery was attained while maintaining negligible tissue heating, even under irradiance levels as high as 1375mW/cm²^17^; subsequently, they patented it as indicated in the current patent application from a database^28^. These prior works have demonstrated impressive OWPT capabilities across a wide range of topics, e.g., high-power NIR-II charging, PV stimulation platforms, subdermal solar harvesting under tissue or skin, and so forth.

To establish an optical link for transcutaneous optical applications such as OWC and OWPT, selecting appropriate wavelengths is essential, as biological tissue is a complex medium that scatters and absorbs light. Hemoglobin, which is abundant in blood-rich regions, tends to attenuate light penetrating biological tissue^29^, particularly visible light^30^. Near-infrared (NIR) light is gaining increasing interest due to its broad range of applications in biomedical fields. It is known to penetrate the superficial layers of the biological tissue with relatively low absorption and scattering compared to other wavelengths^22^. Wavelengths in the NIR I range (~ 750–850 nm) exhibit optimal optical properties for penetrating multiple layers of biological tissue^31^, making them particularly suitable.

### Combining communications and WPT using light

OWC combined with OWPT offers a practical approach to enable data transmission while simultaneously replenishing onboard energy storage without the need for non-invasive power delivery with IEDs. Importantly, most relevant studies have focused on either NIR-based OWC or OWPT, whereas joint implementation of both functions remains relatively underexplored. Interestingly, we previously demonstrated joint optical data and power transfer across a 3 cm-thick ex vivo porcine tissue sample under direct skin illumination, i.e., without any intervening clothing or textile layer. We employed a fully functional hardware setup built from commercial off-the-shelf (COTS) components^32^. An 810 nm NIR LED has been employed as the transmitter. Meanwhile, photodetectors and PV cells were used for data and power reception, respectively. For wireless communication evaluation, a data rate of approximately 95 kb/s was achieved using Gaussian Minimum Shift Keying (GMSK) modulation. For power transfer assessment, ~ 4.2 joules of harvested energy were stored in a supercapacitor over ~ 26 h under continuous exposure. Meanwhile, during concurrent data transmission and power delivery, a small amount of energy was still harvested, thereby validating the feasibility of forward telemetry combined with energy reuse via a shared optical channel.

### Research gap

**Fig. 1:**
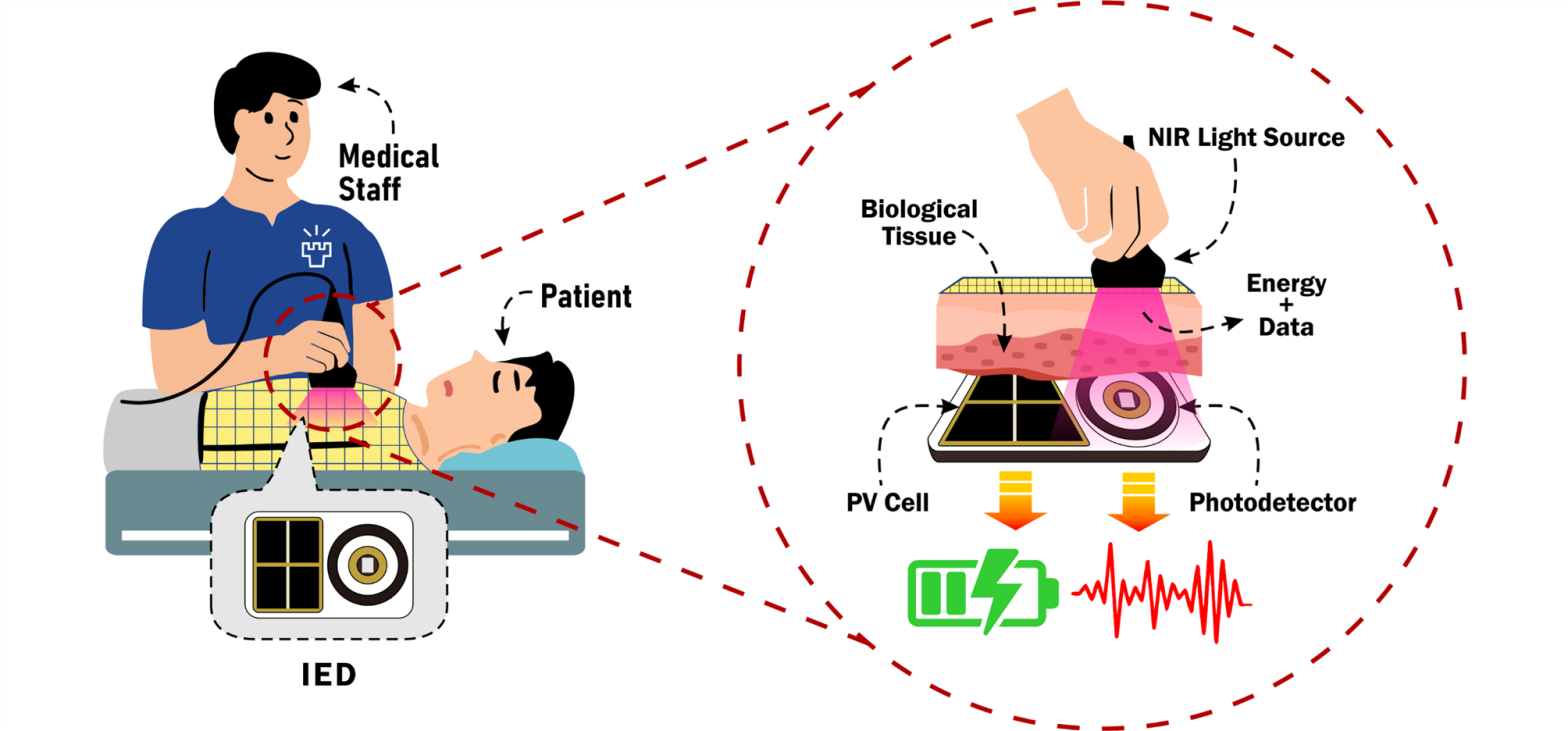
Illustration of a real-world application scenario. A single layer of cloth may cover the IED site. Wearable-to-IED links can be partially blocked by this clothing, leading to increased optical attenuation and potentially impacting system performance.

Furthermore, most reported experiments to date have been conducted under idealized conditions without any intervening textile or clothing layers^33^. In the real-world deployment of such a system, a critical practical challenge arises: clothing obstruction. For example, consider a patient equipped with an IED undergoing optical battery recharging while wearing a cloth (as illustrated in Fig. 1). In this case, the light beam must first pass through the clothing before reaching the skin surface. The presence of clothing introduces additional attenuation, potentially reducing the optical intensity reaching the tissue. Moreover, cloth interaction may alter the power distribution (affecting the beam’s thermal profile) and accumulate heat at the cloth surface, thereby compromising the system’s efficacy and tissue safety. While our previous study has demonstrated the feasibility of (1) OWC, (2) OWPT, and (3) their joint operation through biological tissue^32^, real-world deployment must account for additional barriers, such as superficial clothing layers.

### Novelty

Previous relevant studies have primarily focused on OWC (Table 1), while others have focused on OWPT, as elaborated earlier. Meanwhile, this study demonstrates simultaneous power and data transmission over a single optical link in a realistic deployment scenario. We evaluated the impact of clothing obstruction on system performance, a factor overlooked in other studies. Specifically, we focus on the energy harvesting metrics. Thus, to the best of our knowledge, this is the first study to report the effect of clothing obstruction on joint optical data and power transfer across biological tissue, simulating a realistic scenario in which a patient may wear clothing during active operation, i.e., forward telemetry, while receiving energy delivery. This study employed an 850 nm LED for data and power transmission on a single channel. In the context of a transmitter device for OWC across biological tissue, there are two options: vertical-cavity surface-emitting lasers (VCSELs), as in^11^, and LEDs, as in^5^. While VCSEL sources offer high-speed data transmission due to their narrow beam divergence and high coherence, they may pose greater safety risks, particularly to the retina, given their concentrated emission profile. In contrast, LEDs offer a safer, cost-effective alternative with a wide optical bandwidth. Additionally, LEDs are known for their long operational lifetime, attributed to the use of durable semiconductor materials^5^. In contrast to other studies that used only a photodiode for data reception, this work adopts a dual-receiver architecture that serves dual purposes: a photodiode for data recovery and a PV cell to harvest residual optical energy.

**Table 1:** Benchmark of this study compared to the selected prior studies on OWC across biological tissue operating around the NIR-I region, specifically at 850 nm.

Reference	Wavelength (nm)	Transmitter	Receiver	Tissue thickness (mm)	Telemetry direction	Power transfer scenario	Clothing obstruction scenario
^34^	850	VCSEL	Photodiode	3	Backward	No	No
^35^				2, 4, 6	Backward		
^36^				2, 4, 6	Backward		
^37^				2	Backward and forward		
^38^				2.5	Backward		
^39^				3.5	Backward		
^40^				4	Backward		
^5^		LED		10 + 50 (air gap)	Backward		
^41^			Photodiode	2.3 + 800 (air gap)	Backward		
^42^	850	µLED	Photodiode Tandem PV cell	N/A	Backward	Yes	No
^43^		LED	LED and PV cell	N/A	Backward		
^11^		VCSEL	Photodiode PV cell	3	Backward and Forward		
This study		LED	Photodiode PV cell	40	Forward		Yes

### Forward telemetry definition and proposed implementation scenario

Forward telemetry, as defined in this paper, refers to wireless data transmission from an external unit to an IED, typically used to send specific commands to the IED. It could be doing actions such as: (1) updating/adjusting IED settings (e.g., stimulation parameters, patient ID, firmware, and so on) or (2) administering therapeutic doses, or other similar functions that involve sending commands from an external controller to the IEDs. To this end, secure transmission to prevent unintended activation or command interception is highly required for forward telemetry purposes. Such a feature is particularly relevant for smart IEDs that require external control, e.g., neurostimulators, drug delivery systems, or biosignal-activated actuators.

In this study, we use an optical approach to implement forward telemetry, in which modulated light carries encoded data and energy through biological tissue. The light beam serves a dual function, enabling both data communication and energy delivery to the IED. We employed the same system as in^32^ to simulate a typical forward telemetry scenario, in which a high-resolution image file is transmitted from a host computer to a client computer via a wireless optical link. This image is treated as a representative payload, such as a medical configuration file or stimulation command, intended for the IED. During this data transfer process, the progression of energy accumulation in a supercapacitor is concurrently monitored. This setup mimics a realistic scenario in which optical-based therapeutic instructions and optical energy are delivered simultaneously, passing through biological tissue to an IED. Interestingly, a supercapacitor-based energy storage approach is employed in this study as an alternative to conventional batteries, opening new avenues for safer, long-lasting (i.e., high-energy storage cycle) and biocompatible power solutions in future IED systems^44^.

### Targeted research questions

To sum up, this paper addressed the following research question: How does clothing obstruction affect the performance of joint optical wireless data and energy transmission across biological tissue? Practically, this paper reused the optical system from^32^, as mentioned earlier, to bring it closer to more realistic usage scenarios, i.e., by including clothing. Two test conditions were conducted using the same testbed: (1) the NIR light beam passed directly through an ex vivo porcine tissue sample (no clothing scenario), which serves as the baseline, and (2) a cloth was placed between the NIR source and the tissue (clothing-covered scenario). A comparison was then conducted in terms of the received optical power, voltage output of the PV cell in an open circuit ($\:{V}_{OC}$), and the increase in supercapacitor voltage. While this work does not aim to fully characterize all textile effects, it provides a practical initial benchmark that shows how a typical clothing layer can influence the performance of an end-to-end optical system.

## Methods

### Experimental setup

**Fig. 2:**
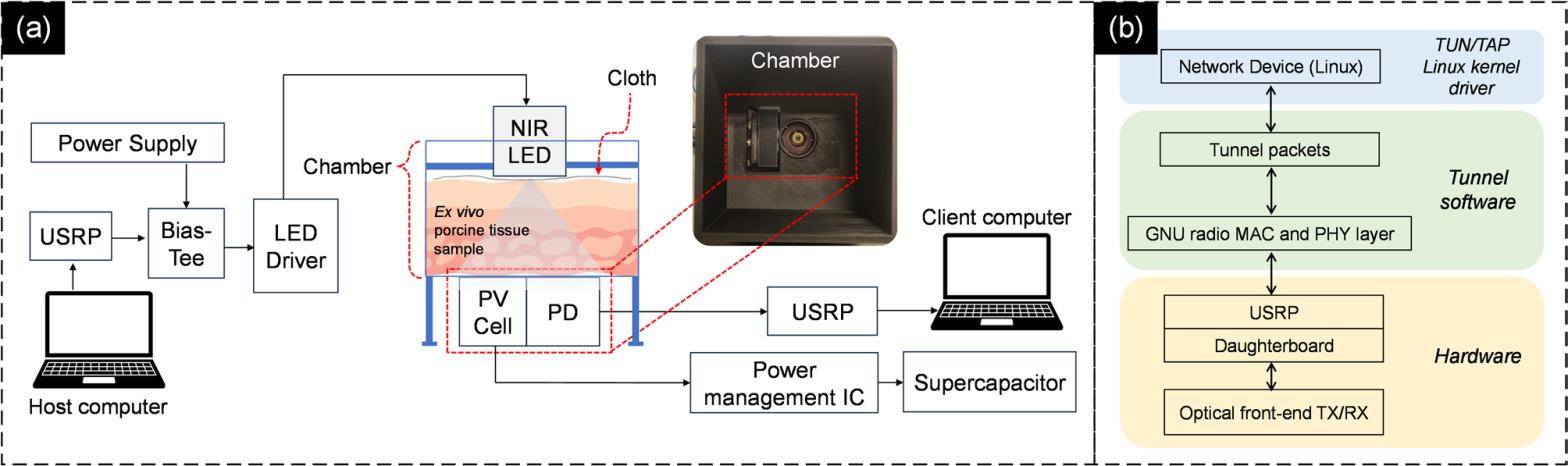
(**a**) The setup integrates a host and a client computer for control and data acquisition, USRP transmitter and receiver modules for signal transmission and reception, and optical front-end transmitter and receiver. The optical front-end transmitter includes an LED driver, the NIR LED, and a Bias-Tee for signal conditioning. The optical front-end receiver consists of a PV cell connected to a PMIC for harvested energy control and a photodetector. The photodetector and PV cell are positioned to receive illumination from an NIR LED, directed through an ex vivo porcine tissue sample to simulate biological conditions. A supercapacitor is monitored via a dedicated computer to display the charging progression. Real-time voltage rise is captured using a Piolog oscilloscope; (**b**) The system begins at the network layer with a virtual TUN/TAP interface provided by the Linux kernel, which generates raw IP packets. These packets are passed to the Tunnel software layer, where they are processed for transmission. The GNU Radio MAC and PHY layers handle the modulation and encoding of the packet stream. The signal is then passed to the USRP hardware, which consists of a baseboard and a daughterboard, serving as the digital front end. The modulated signal is transmitted through an optical front end. This architecture enables IP-based data transfer over an optical link, forming a complete communication tunnel through light.

The experimental objective was to assess how cloth affects the performance of joint optical data and power transmission to the IED. We employed a testbed which was implemented from commercially available components, as illustrated in Fig. 2a. The system architecture comprises two subsystems: an external transmission unit and an internal receiver unit.

The external transmitter includes the Universal Software Radio Peripheral (USRP) 2920 software-defined radio (SDR), configured with GNU Radio, which modulates the outgoing data stream using GMSK. The modulated signal is fed into a Bias-Tee (Mini-circuits ZFBT-4R2GW-FT+) to superimpose a DC offset required for powering the NIR LED (Thorlabs M850LP1). The external power supply (Agilent E3630A) was used to provide the required DC offset of ~ 1 V. The LED driver (Thorlabs DC2200) is set to “external modulation” mode, allowing the NIR LED to emit both continuous light and the modulated data. Under this mode, the LED driver provides 400 mA per 1 V from the external input to the NIR LED. Suppose the external power supply offers 0.5 V; thus, the LED driver will generate 0.5 V to the NIR LED.

The internal receiver unit mimics an IED system and consists of two subsystems. A photodetector (Thorlabs PDA36A-EC) was used to receive the incoming optical signal, capture it, and forward it to the receiving USRP for demodulation. Simultaneously, a monocrystalline silicon PV cell (IXOLAR SM141K04LV R3.5), positioned near the photodetector, was used for energy harvesting, which absorbs the residual optical energy and feeds it to a power management integrated circuit (E-Peas AEM10330), which in turn charges a 0.16 F supercapacitor (CAP-XX GW209+). This configuration allows the IED to receive both control data and power wirelessly, using a shared optical path. The PV cell has characteristics as follows: (1) Area: 6.02 cm², (2) PV cell’s $\:{V}_{OC}$ maximum: 2.76 V, and (3) PV cell’s short-circuit current ($\:{I}_{SC}$) maximum: 46.7 mA. Meanwhile, the PMIC settings are as follows: (a) MPPT at 80% of $\:{V}_{OC}$ and (b) setting of a supercapacitor threshold: 1–4.5 V range.

Optical links are typically directional and require precise alignment for optimal performance. Therefore, in this study, we concentrated on the aligned configuration scenario for two purposes: creating dependable baseline measurements and ensuring accurate evaluation. In this study, we employed a chamber previously introduced in^45^. The testbed facilitates precise vertical alignment between a mounted NIR LED and a photodetector across an ex vivo porcine tissue sample, ensuring consistent optical coupling and minimizing possible misalignment. As shown in Fig. 2a, the mounted NIR LED was fitted into the top part of the testbed, while the biological tissue was placed inside a cubic enclosure designed to house the sample. This enclosure also functions as a light-shielding chamber, minimizing interference from ambient illumination and allowing experiments to be conducted under standard laboratory lighting conditions. The mounted NIR LED pad was insulated to prevent short-circuiting when in contact with moist biological materials. The photodetector was installed at the bottom of the enclosure, with the vertical optical path pre-aligned to the LED aperture above, ensuring automatic alignment without manual adjustment. Additionally, two protruding poles at the top serve as hand grips to facilitate manual pressing and releasing the LED against the sample.

### Communication and data protocol

To establish a functional forward telemetry channel, a virtual network tunnel is configured between host and client computers using the TUN/TAP interface in GNU Radio. Figure 2b illustrates the system’s structure. The data stream is transmitted using the User Datagram Protocol (UDP), which facilitates low-latency, connectionless delivery. Once communication is verified via ping, a high-quality image (in .TIFF format) is transferred from the host to the client using the *sudo cp.* command, routed through the optical path. The image transmission in this experimental case acts as a substitute for actual medical command data that could be uploaded to an IED. Throughput and packet loss metrics have been evaluated using *iperf3* to establish a reliable wireless link across the biological tissue channel.

### Propagation medium (ex-vivo porcine tissue and clothing)

Fresh ex vivo porcine tissue samples, approximately 4 cm-thick, are used to simulate the human tissue, as their optical (i.e., scattering and absorption coefficient), structural (e.g., skin, fat, and muscle), and anatomical (e.g., hairless skin) properties closely resemble those of human tissue^46^. In general, ex vivo porcine tissue is a widely used model for initial biomedical studies, offering a more accessible and reproducible approach than in vivo testing (animal models or human participants). The use of ex vivo porcine tissue is ethically acceptable, enabling controlled laboratory experiments on optical transmission through biological material^47^, especially useful for large-scale studies or experiments that require fresh samples. Specifically, a study has shown a strong positive correlation in optical parameters between pig and human tissues^46^, supporting their use as models for optical techniques, including OWC and OWPT, and their joint application.

**Fig. 3:**
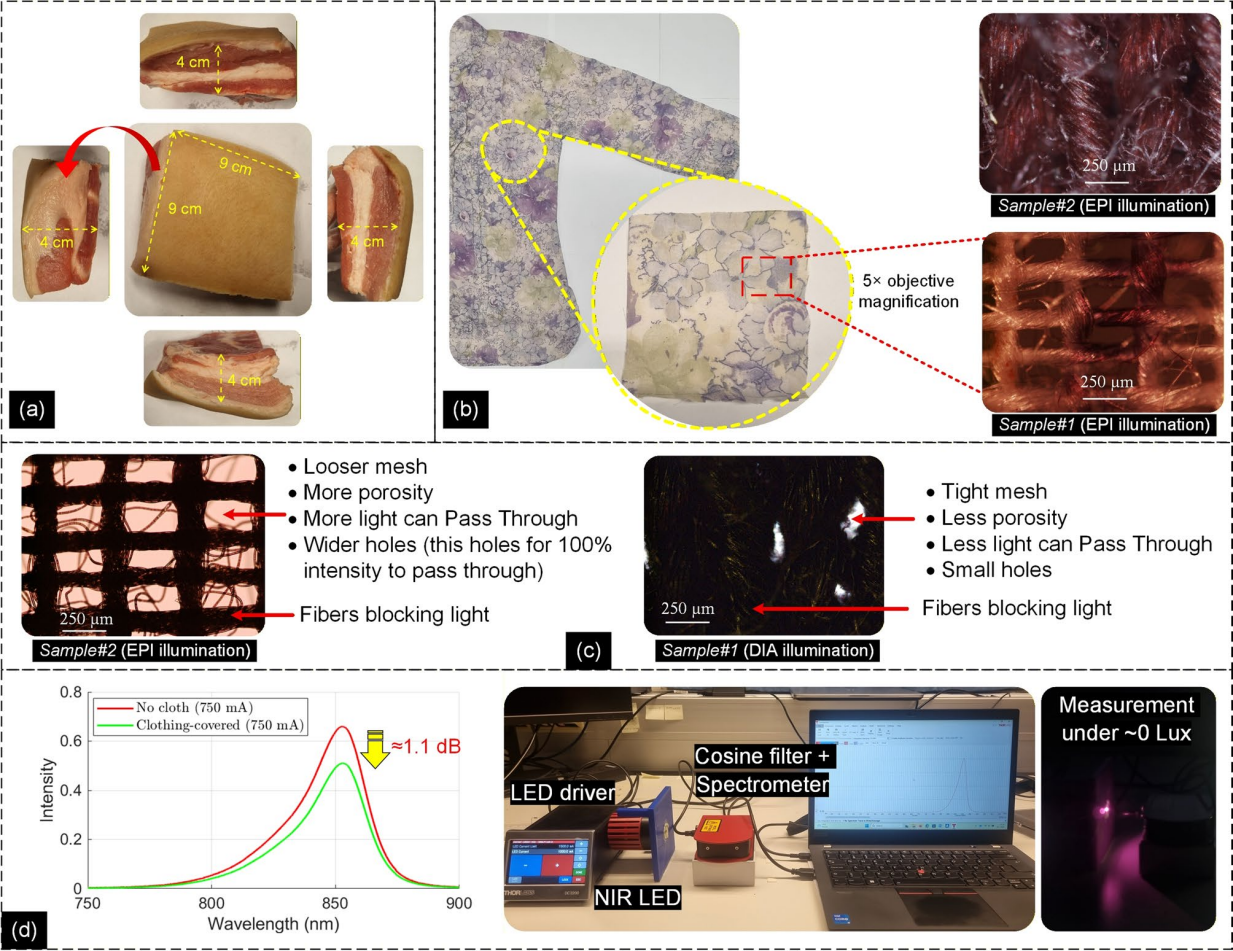
(**a**) A fresh ex vivo porcine tissue sample used in this study, the tissue sample consisted of both muscle and fat layers, with varying thickness depending on the orientation. For example, one side of the sample was predominantly muscular, while the opposite side contained a higher proportion of fatty tissue; (**b**) Representative textile samples used in this study; (**c**) Microscopic image of each textile sample, showing a small and highly porous structure, showing on DIA illumination. A stage micrometer with 10 μm divisions was used to calibrate the pixel size at each magnification, enabling quantitative measurement of pore size and yarn width in physical units; (**d**) spectral analysis of NIR LED on no cloth settings and covered by a *Sample#1*, where the measurement was taken at a 5 cm distance in free space using specialized software (ThorSpectra v1.3.0/1.0.0). The NIR LED was driven at 750 mA.

However, the use of ex vivo samples should be considered an approximation rather than a direct substitute for real human tissue, as variations in composition, hydration, structural heterogeneity, and pigmentation may affect optical attenuation. To this end, the study offers experimentally grounded insights into realistic scenarios, with direct quantitative relevance to human in vivo conditions, which should be interpreted cautiously. The NIR light is directed in a “skin-first” orientation, mimicking transcutaneous exposure. The exposed surfaces of the samples are larger than 6 × 6 cm to prevent light leakage at the edges (Fig. 3a). Importantly, ex vivo porcine tissue may change optical properties over time due to dehydration, prolonged exposure to room temperature, and NIR illumination, making it difficult to directly compare different textile conditions unless each measurement is conducted under carefully controlled, identical conditions. To minimize this bias, the study focused on a limited, controlled comparison between the no clothing setting and clothing-covered cases within the same experimental framework.

This study considers two cotton-based textile samples with different thicknesses, representing distinct attenuation scenarios, labelled Textile *Sample#1* and *Sample#2* (Fig. 3b). Specifically, *Sample#1* is characterized by a white color, a very thin fabric layer (~ 0.25 mm thick), and a patterned surface rather than a uniform white tone. Meanwhile, *Sample#2* is a thicker fabric layer (~ 1 mm) and a dark color. Importantly, using *Sample#1* allows us to evaluate whether even minimal, seemingly non-intrusive fabric obstruction can measurably affect the performance of joint optical wireless power and data transmission. Each cloth was cut to conform to the tissue area, measuring approximately 10 cm × 10 cm.

We first characterize both cotton-based textile samples using an optical microscope (Nikon ECLIPSE LV 100) equipped for bright- field imaging to observe their microstructure. We acquired images under both episcopic (EPI, reflected light) and diascopic (DIA, transmitted light) illumination modes at 5× objective magnifications. The EPI mode emphasizes yarn morphology, surface roughness, and crossing points. In contrast, DIA imaging reveals the arrangement of yarns and, crucially, the inter- yarn gaps that form the porous network through which NIR light can pass. Image capture and basic exposure control were performed using the NIS-Elements software (version 3.22.3.22.11). As shown in Fig. 3c, *Sample#1* exhibits higher “open” regions—corresponding to inter- yarn holes with high transmitted intensity—from “closed” regions occupied by yarns. Meanwhile, *Sample#2* is vice versa.

Second, the spectral characteristics of the textile sample under 850 nm NIR light are observed to determine whether the emission wavelength shifts. Textiles are highly variable because they depend on multiple factors, including material composition, thickness, layering, color, weave density, porosity, texture, and moisture content. Owing to this complexity, in particular, it has attracted significant research attention across disciplines, including engineering (e.g., textile optics). More specifically, textile optics has been widely explored for a range of applications, including light-protective clothing^48^, stealth and camouflage^49^, light therapy^50^, and imaging through clothing layers^51^. Previous studies have shown that dye structure, dye concentration, and dyeing conditions can significantly affect optical transmission^48,49^. In addition, structural properties such as porosity, thickness, moisture content, and surface texture strongly influence the amount of transmitted NIR light^3^. Collectively, these studies provide useful spectral characterization for understanding how textiles transmit light across a broad optical range, including the UV, visible, and NIR (UV-Vis-NIR) regions. In this work, the joint optical data and power transmission system was designed around a fixed operating wavelength of 850 nm, selected for its favorable tissue penetration properties compared with other wavelengths, e.g., UV and visible light. After fixing the wavelength, the effect of the selected textile samples was evaluated at the system level. Based on our interpretation of the spectral data reported in^48–51^, when the optical source operates at a single wavelength or within a narrow spectral band, the dominant textile effect is primarily attenuation at that wavelength. This interpretation is consistent with the experimental spectra in Fig. 3d. Specifically, the figure compares operation with no textile layer and the clothing-covered case at 750 mA, using *Sample#1* as the representative textile layer. Spectral measurements using a spectrometer (Thorlabs CCS200/M) with a cosine corrector (Thorlabs CCSB1). The result shows that both spectra remain similar in shape at the same drive current, but with lower intensities. This indicates that the textile layer mainly reduces the optical intensity rather than shifting the spectral center of the LED emission (Fig. 3d).

### Measurement methodology

The physical alignment of all components in the testbed, including the tissue sample, photodetector, and PV cell, was maintained at the baseline configuration (i.e., no clothing layer). The only variable altered was the insertion of the cloth layer directly on top of the ex vivo tissue sample, covering the NIR LED beam path. The data obtained in this comparative study are as follows:

- Firstly, we measured the received optical power metric. We used an optical sensor (Thorlabs S121C) integrated with an optical power meter (Thorlabs PM100D) to measure optical power after it passed through an ex vivo porcine tissue sample under two conditions: no clothing layer and clothing-covered with a single layer of cloth. The LED current is varied from 150 mA to 750 mA via the LED driver in constant-current mode. At each setting, the measuring result is recorded. The optical power meter’s setting is described in^52^.
- Secondly, we measured the PV cell’s $\:{V}_{OC}$ metric. We used a digital multimeter (Klein tools MM400) to measure $\:{V}_{OC}$ after light passed through the same sample under the same two conditions. $\:{V}_{OC}$ serves as a practical indicator of the amount of light reaching the PV cell at varying NIR LED intensities.
- Thirdly, we measured the supercapacitor voltage progression metric under two scenarios, i.e., with and without clothing conditions. The LED driver is operated in an external modulation mode. The same high-resolution image file (approximately 7 MB) was transmitted from the host to the client computer to simulate forward telemetry. The experiment simulates a forward telemetry event lasting approximately 10 min, during which various actions may be performed, such as changing the device ID, administering therapeutic doses, updating firmware, or transmitting specific stimulation data to the IED. During data transmission (forward telemetry event), the progression of the supercapacitor voltage was recorded for each scenario (seven trials per scenario) using a digital oscilloscope (PicoScope 3405D series). The AEM10330 PMIC manages supercapacitor charging. The supercapacitor voltage profile is recorded throughout the 10-minute image transmission period, representing both the data and power operations. The energy stored in the supercapacitor ($\:E$) is calculated using (1), where $\:{V}_{max}$ and $\:{V}_{min}$ are the final and initial voltages across the supercapacitor, respectively, and $\:C$ is the capacitance of the supercapacitor (i.e., 0.16 F). Comparing the supercapacitor charging progression in both scenarios provides insights into the impact of clothing obstruction on energy-harvesting performance.

1 $$\:E=\frac{1}{2}\times\:C\times\:\left({V}_{max}^{2}-{V}_{min}^{2}\right)$$

It should be noted that in this study, communication performance was evaluated at the end-to-end link level using the previously developed UDP-over-TUN/TAP optical architecture^32^. The quality of forward telemetry was evaluated using fixed-size image transmission, as previously mentioned, focusing on transfer time and average throughput. Specifically, a TIFF image file was chosen as the payload because it offers a reproducible, lossless, and medically relevant data format widely used in biomedical imaging workflows. Communication reliability was examined based on transfer success, throughput consistency, and packet-loss behavior under both the absence and the presence of an intervening textile layer. Importantly, no packet loss or link interruption was observed under the tested conditions. Once the photodiode receives sufficient NIR irradiance to maintain the optical tunnel connection, the NIR LED data is sent to the photodiode and waits for successful reception. Therefore, under the present experimental conditions, the main communication impact of clothing was observed in transfer performance rather than link failure.

Ensuring the safety of the implant and surrounding tissue is of utmost importance in this study. In general, heat accumulated near the implant is dissipated through the surrounding tissue and skin, together with metabolic heat regulation^53^. Accordingly, each telemetry session was limited to ~ 10 min to provide a cooling interval before subsequently irradiating the tissue surface by the 850 nm NIR LED. Our system consistently maintained a transmission session of ~ 10 min, as our intended, with an average transfer rate of about 11 KB/s. Table 2 shows the forward telemetry profile applied in this study. A comparable exposure duration of ~ 10 min (irradiance of 0.3 W/cm^2^ under NIR-II: 1000–1350 nm) has also been reported in another study^23^.

**Table 2:** Forward telemetry parameters used in this study.

Parameter	Description
Functions	Administering therapeutic doses or changing the IED configurations
Transfer rate (KB/s)	~ 11 KB/s
Telemetry duration (s)	10 min
Medical payload	TIFF image (~ 7 MB)
Dose	7× repeated transmission (Trial I – VII)

## Results and analysis

### Received optical power under no cloth and clothing-covered scenarios

**Fig. 4:**
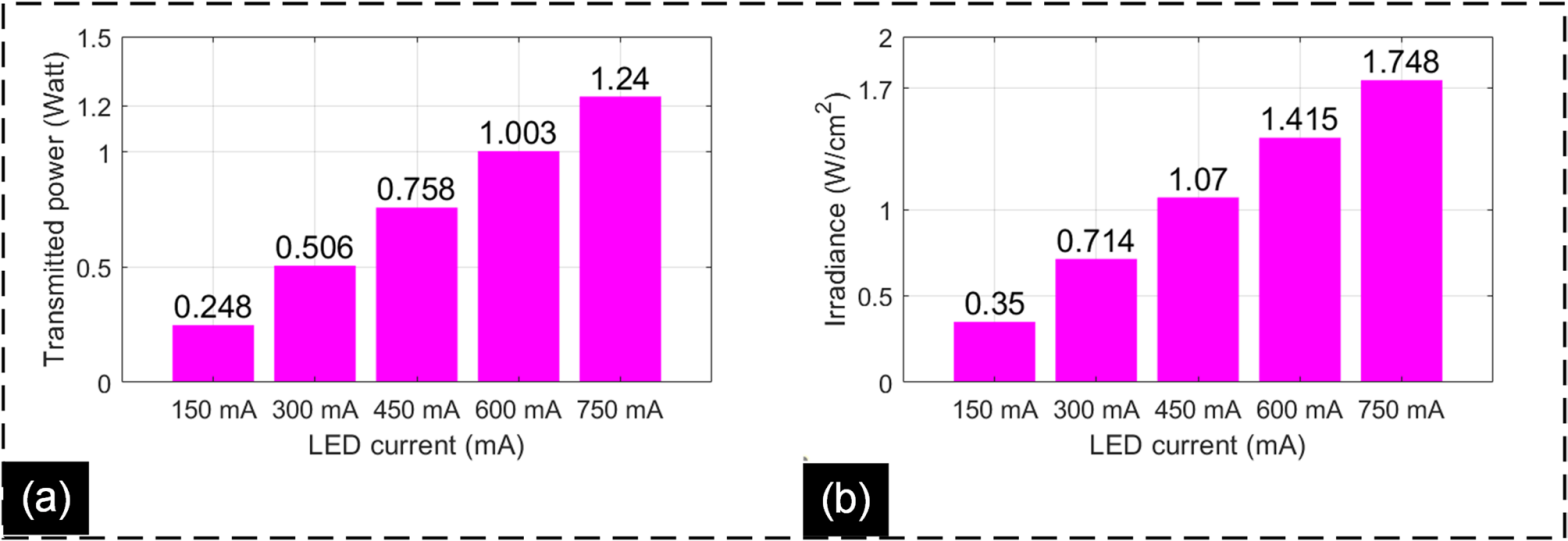
Measurement results for the incident power of the NIR LEDs used in this study.

Figure 4 shows the output characteristics of the Thorlabs M850L4 NIR LED driven at currents ranging from 100 mA to 500 mA. The transmitted optical power (in mW) and corresponding power density (in mW/cm²) were measured at a distance of 0.1 cm using an optical power meter PM100D. For this study, we selected a single operating point, specifically the “external modulation” mode, in which the NIR LED was biased at 1 V via an external power supply, yielding a current of 400 mA and an optical output of 142 mW (200 mW/cm²).

Afterward, we measured the received optical power of NIR light at 850 nm (single operating wavelength) after it passed through a 4-cm thick ex vivo porcine tissue sample (a single tissue thickness) to quantify the effect of clothing. The NIR LED source was configured at different power levels by varying the drive current to 150, 300, 450, 600, and 750 mA in constant-current mode. We also bias the NIR LED via an external modulation mode, with 1 V supplied by an external power supply (equivalent to 400 mA).

**Fig. 5:**
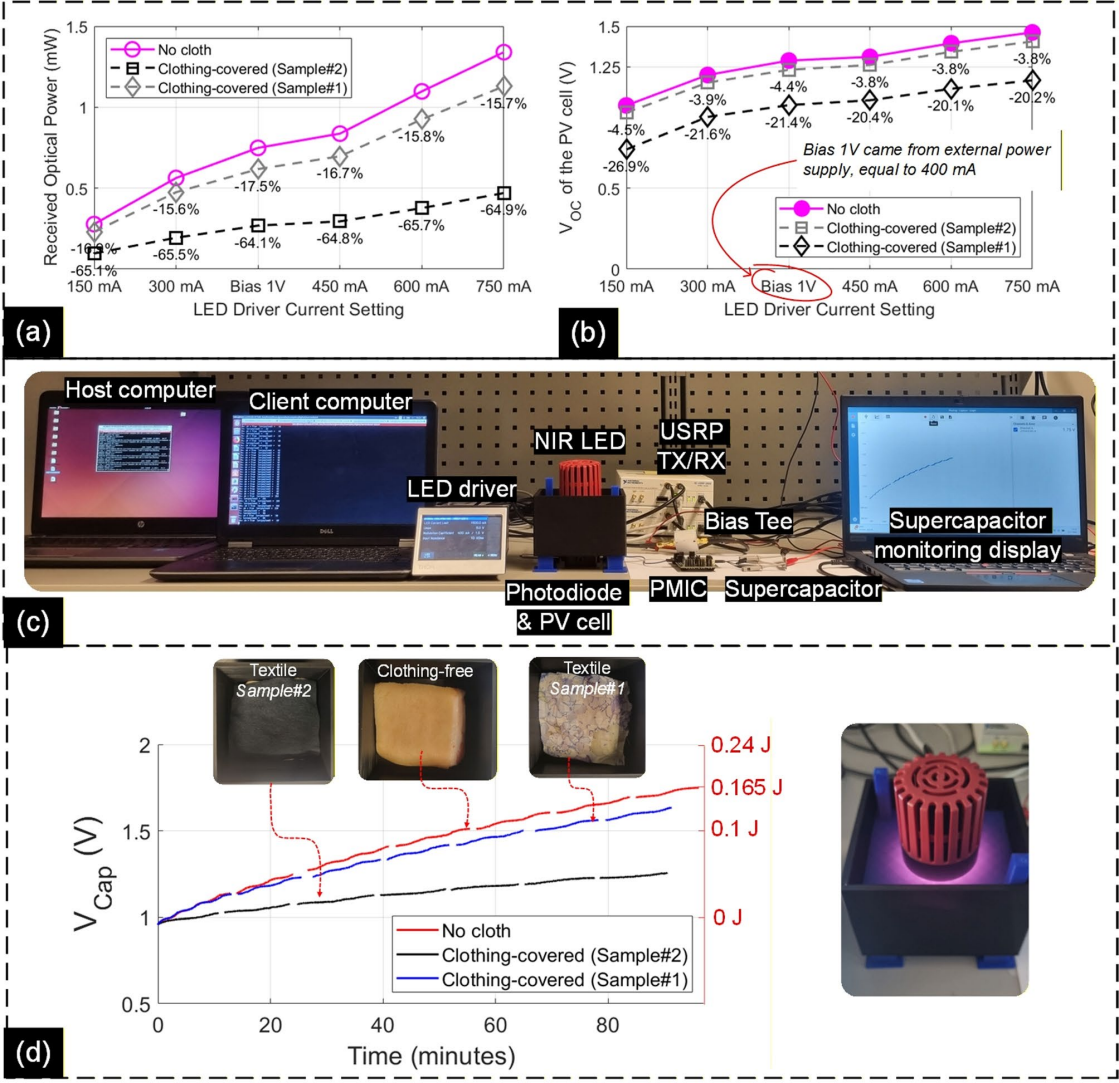
(**a**) Comparison of received optical power through a 4 cm-thick ex vivo porcine tissue sample under clothing-covered and no cloth conditions. The graph illustrates the impact of clothing obstruction on transmittance; (**b**) Comparison of $\:{V}_{OC}$ of the PV cell after light transmission through a 4 cm-thick ex vivo porcine tissue sample, under clothing-covered and no cloth conditions. The graph highlights the impact of clothing obstruction on PV cell output; (**c**) laboratory setup demonstrating joint data and power transmission across biological tissue; and (**d**) Supercapacitor charging progression during forward telemetry through a 4 cm-thick ex vivo porcine tissue sample, comparing clothing-covered and clothing-covered conditions. The graph demonstrates the impact of clothing obstruction on the supercapacitor voltage ($\:{V}_{Cap}$) and energy stored in the supercapacitor.

The temperature of the fresh ex vivo porcine sample at the time of purchase from the market was approximately 11 °C. The reading results (Fig. 5a) demonstrate a clear reduction in received optical power when clothing is present across all input power levels. *Sample#1* exhibits minimal optical absorption (high transmittance), making it less obstructive to light. than *Sample#2*. For instance, on *Sample#1* at an NIR LED output of 150 mW, the received optical power in the no cloth case (i.e., 0.278 mW) was higher than in the clothing-covered case (i.e., 0.231 mW), resulting in an approximately 16.90% drop. The average drop across all NIR LED settings for *Sample#1* was 16.38%, whereas the black cloth produced a much stronger average reduction of about 65.0% relative to the no cloth case. The data confirm that even a single layer of a thin cloth, i.e., *Sample#1*, can considerably attenuate NIR light before it reaches biological tissue. In contrast, a thicker cloth can worsen this effect.

An effective attenuation model can be expressed as (2), where $\:{P}_{tx}$ is the transmitted optical power, $\:d$ is the tissue thickness, and $\:{\mu\:}_{\mathrm{e}\mathrm{f}\mathrm{f}}=\sqrt{3{\mu\:}_{a}({\mu\:}_{a}+{\mu\:}_{s}^{{\prime\:}})}$. For fixed tissue optical properties, attenuation with tissue thickness is expected to follow an exponential trend, based on (2). However, when tissue thickness and optical properties are fixed, $\:{P}_{rx}$ becomes proportional to $\:{P}_{tx}$, so varying $\:{P}_{tx}$ produces approximately a linear change in $\:{P}_{rx}$. Therefore, the measurement result shown in Fig. 5a is assumed to be consistent with the approximation of (2).

2 $$\:{P}_{rx}={P}_{tx}\mathrm{exp}\left(-{\mu\:}_{\mathrm{e}\mathrm{f}\mathrm{f}}\:d\right).$$

###  $V_{{oc}}$ of PV cell across clothing-covered scenario

We then removed the sensor S121C and replaced it with the receiver (photodetector and PV cell), and measured the $\:{V}_{OC}$ of the PV cell placed behind the ex vivo porcine tissue. The PV cell was illuminated through the same porcine tissue sample under the same five NIR LED power levels. The results reveal a strong correlation between the LED drive power and the resulting $\:{V}_{OC}$ across both scenarios (Fig. 5b). As expected, PV cell output, in general, is increased with higher illumination levels. However, across all corresponding power settings, the clothing-covered configuration yielded consistently lower $\:{V}_{OC}$ values than the no cloth case. This reduction can be attributed to a diminished photon flux reaching the PV surface due to the cloth’s absorptive properties. *Sample#1* caused a relatively modest reduction in $\:{V}_{OC}\:$for all NIR LED settings relative to the no cloth case, with an average decrease of about 4.04%. In contrast, Sample*#2* caused a much stronger reduction, averaging about 21.8%. Despite this drop, the PV cell still produced voltages within the PMIC’s operable input range (i.e., at least 100 mV), indicating partial energy-harvesting viability even under obstructed conditions, albeit with a trade-off in supercapacitor charging duration.

### Supercapacitor charging under No cloth and clothing-covered scenarios

The LED driver is then connected to the USRP transmitter, and the photodetector is connected to the USRP receiver to demonstrate forward telemetry and supercapacitor charging (Fig. 5c). As elaborated above, an image file (i.e., 7136 KB.TIFF) was transmitted from an external host computer to a receiving unit using a modulated 850 nm optical link. Simultaneously, the energy harvested from residual illumination was stored in a supercapacitor and monitored throughout the transmission session.

Figure 5d compares the supercapacitor charging profiles under no cloth and clothing-covered (i.e., using *Samples#1* and *#2*) conditions. As expected, the supercapacitor voltage increased incrementally during each transmission session. For instance, under the no cloth condition, in Trial I, the voltage rose from 1.00 V to 1.14 V, and subsequently, in Trial II, it further increased from 1.14 V to 1.27 V, and so forth. It requires 96.08 min to go from 0.964 V to 1.750 V. However, the supercapacitor exhibited a decrease in voltage under the clothing-covered case. Compared to the no cloth condition, *Sample#1* experienced a moderate decrease in charging speed, with an average reduction of approximately 9.4%. In contrast, *Sample#2* exhibited a significantly larger slowdown of around 59.9%. The final energy stored in the supercapacitor after seven data transmission cycles was 0.165 Joules, 0.132 Joules, and 0.046 Joules in the baseline test (no clothing obstruction) and in the clothing-covered case (*Sample #1*) and *Sample#2*, respectively. Compared with the no cloth condition, energy decreased by about 19.2% and 71.9% for *Samples#1 and #2*, respectively.

The study demonstrates that modulated light, originally designed solely for data transmission, can also be used for energy harvesting. The primary idea of the proposed system is to capture the part of the optical signal carrying data that passes through biological tissue and convert it into electrical energy using an implanted PV cell. This method enables both data and power transmission using a single optical channel (i.e., through an NIR LED). After extended experimentation at room temperature (~ 7 h), the tissue sample temperature was approximately 22 °C.

The clothing layer exhibits absorptive properties that introduce additional optical attenuation, thereby reducing the received optical power and the PV cell’s $\:{V}_{OC}$. This results in a lower final supercapacitor voltage and slower charging behavior compared to the case without an intervening textile layer. The results demonstrate that even a thin layer and low porosity of clothing can still contribute to optical power attenuation, negatively impacting energy harvesting. Accounting for everyday constraints when considering an additional feature for IED applications, such as energy harvesting, is crucial. Of course, removing optical obstructions, such as clothing above the skin, enhances link performance. Nonetheless, since clothing is usually unavoidable in practical wearable-to-implant applications, a more relevant future goal is to ensure the optical link functions effectively despite any type of clothing-induced attenuation.

## Discussion

### Limitations on the variety of ex vivo porcine tissue

This initial study did not involve human subjects. Instead, ex vivo testing with biological tissues was used to ensure ethical compliance and is likely more practical while still accurately simulating the optical properties of human tissue. Since we conducted the experiment for ~ 7 h, there may be changes in the tissue’s optical properties due to moisture loss after prolonged exposure to room temperature. Accordingly, a post-experiment evaluation was performed on the ex vivo porcine tissue to assess data reliability and quantify the degree of deterioration resulting from prolonged exposure to the temperature-room conditions. We measured the received optical power and compared data taken before (a fresh sample) and after extended measurement campaigns (post-experiment).

**Fig. 6:**
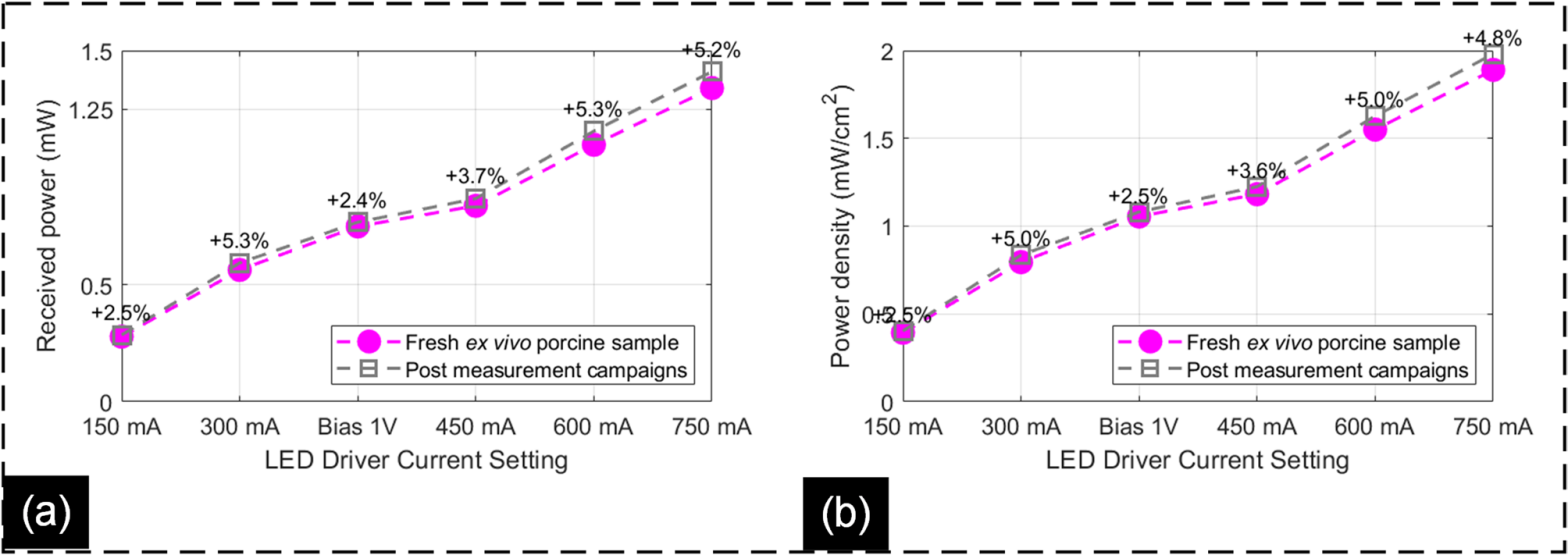
Comparison of the data between before (with a fresh ex vivo porcine sample) and after an extended measurement campaign duration (post-experiment), which is ~ 7 h: (**a**) received optical power; (**b**) power density.

The results, summarized in Fig. 6, showed that all values increased by a noticeable amount (ranging from 2.4% to 5.3%). Measurements taken after ~ 7 h of measurement campaigns showed increased received optical power compared with baseline data collected under fresh sample conditions. We attribute this to increased tissue transparency resulting from structural changes that occurred as the sample dried (i.e., tissue dehydration over time, which makes it more optically transparent). The measurement results are available in the Supplementary file.

These results highlight the inherent challenges of using biological tissue models for long-duration optical experiments. Specifically, the sample emits unpleasant odors if not properly preserved^52^. While ex vivo pork tissue remains valuable for assessing data transmission, particularly when short experimental times are sufficient, it proves less practical for scenarios that demand prolonged measurement sessions. The comparison data confirms a study conducted by^54^ on optical wireless power transmission across pig skin. Their skin tissue sample progressively lost moisture over time, leading to dehydration that deteriorated its optical properties. As water, an inherent NIR absorber, decreased, the tissue became more transparent, increasing light transmittance. To address this limitation, optical phantoms present a promising alternative for future similar studies. These synthetic tissue models offer long-term durability, supporting extended measurement campaign durations, which are essential in such studies where consistent optical properties are critical.

It should also be noted that this study did not include real-time temperature monitoring nor considering the misalignment setting. For future research, it is essential to implement dual-sample setups (exposed versus control) to validate thermal safety. This study establishes a reliable baseline for analysis, ensuring that any observed performance changes can be attributed to clothing obstruction. For this reason, this study employs a precisely aligned optical setup. Simulating misalignment in scenarios where the conditions are clothing-covered could be beneficial in future studies.

### Sunlight-assisted optical charging

Sunlight is a natural light source that may have a positive impact not only on health but also on engineering. From a health perspective, sunlight is widely known to have beneficial effects. In particular, exposure to natural light plays an important role in human well-being^55^. Spending time outdoors has often been associated with positive effects, especially in improving vitality^56^, i.e., better physical and mental energy. From an engineering perspective, it brings to the idea of sunlight-assisted optical charging as a complementary approach to the controlled, skin-adjacent NIR transmitter (wearable optical transmitter) discussed here. Under this scenario, sunlight could be exploited as an opportunistic supplementary energy source for IED charging during outdoor activities (i.e., so-called photoelectronic-charging^14^), while controlled indoor IED operation would continue to rely on a wearable optical transmitter. A seminal study demonstrated that a sunlight-powered pacemaker, with proper energy storage, could sustain pacing for 24 h with just a few minutes of direct sunlight on an implanted solar cell, proving the potential to power IEDs^57^.

The main drawback of sunlight-assisted optical charging is that it is an uncontrolled source whose intensity varies strongly with time of day, weather conditions, and season^14,58^. In addition, realistic deployment must consider that people spend more time indoors due to urbanization and industrialization, which naturally limits everyday use^59^, making it difficult to predict the duration of direct sunlight exposure. Clothing further complicates this scenario by acting as an intensity attenuator and, in some cases, an additional spectral filter, reducing optical power before the solar radiation reaches the skin, as observed by^50^ through informal measurements. In particular, denser cloths such as jeans and similar pant materials transmit only a small portion of the NIR component and block most of the visible spectrum. Therefore, although sunlight contains NIR wavelengths that may contribute to transcutaneous energy harvesting, the combined attenuation from clothing and tissue makes sunlight-based charging less predictable and potentially impractical for deep IED operation. At the same time, patients naturally prefer to remain clothed during outdoor daily activities. This reason makes sunlight-assisted optical charging may still be relevant as a comfort-oriented supplementary scenario rather than a primary charging strategy. Furthermore, sunlight contains ultraviolet (UV) components in addition to NIR, which raise two issues for such practical applications: energy transfer efficiency and safety considerations. In relation to UV radiation from sunlight, chronic or excessive exposure is a recognized cause of adverse effects, e.g., photoaging^30,60^, and skin damage (increased skin-cancer risk)^61^.

Meanwhile, the wearable-to-implant NIR link employs a controlled NIR LED, enabling more precise control of exposure time, irradiance, alignment, and modulation, which are beneficial for OWPT, OWC, and their joint operation. Nevertheless, it is worth exploring in future studies for sunlight-assisted optical charging, focusing on spectral transmittance at no cloth and clothing-covered scenarios, penetration depth, and thermal effects. Indoor illumination from general lighting infrastructure may also contribute to optical charging in IEDs. However, this contribution is expected to be substantially smaller than that of sunlight, since typical indoor lighting contains mostly visible light content with a lower NIR component, thereby limiting its penetration depth. The attenuation from skin restricts the received optical power available at the implanted PV cell surface.

## Conclusion

Before the present investigation of clothing obstruction effects, joint optical data and power transfer for IEDs had rarely been explored. This study demonstrated joint optical wireless data and power transfer to IEDs using a single NIR light source, accounting for the presence of clothing, which becomes a critical system deployment consideration for the next step clinical adoption. The approach adopted the system structure from previous work, which is a fully functional hardware setup. The study was validated through ex vivo experiments using 4-cm-thick porcine tissue to simulate realistic biological propagation conditions. We conducted forward telemetry based on NIR light and power reuse during data transmission to charge the supercapacitor. It should be noted that the current experiment is at the proof-of-concept stage on the effect of clothing obstructions, and the joint optical data and power transfer system of this study is not yet ready for clinical trials. Specifically, the case-specific experimental evaluation presented in this study, using two representative textile layers, should therefore be interpreted as an initial practical benchmark rather than a comprehensive characterization of clothing effects. Experimental comparisons between clothing-covered and no cloth (i.e., baseline case) conditions revealed that the presence of even a single thin dress layer reduced the received optical power and lowered the PV cell output, thereby degrading the joint optical data and power performance, i.e., slower supercapacitor charging during a forward telemetry event. Nevertheless, the system remained functional under clothing, albeit with reduced efficiency, highlighting both the potential and limitations of simultaneously using NIR light for in-body communication and wireless charging in real-world scenarios.

In future work, it is imperative to investigate the use of multiple textile materials and thicknesses, involve misalignment, and implement real-time thermal monitoring. The impact of clothing color should be further analyzed by evaluating fabrics in different colors, such as red, green, blue, yellow, and grey, since each color may exhibit distinct optical absorption, reflection, and scattering characteristics in the NIR. These differences may significantly affect light penetration and, consequently, the performance of joint data and power transmission across biological tissue. Importantly, ex vivo porcine tissue can undergo optical changes over time, such as dehydration and temperature effects, which can change its absorption and scattering properties. Consequently, using a single biological sample for extensive multi-fabric comparisons might introduce extra variability. Optical phantoms should be considered a substitute for an ex vivo porcine sample, as long-term measurement campaigns require consistent optical properties over time. They offer greater repeatability and are better suited to the systematic investigation of a multi textile types.

## Supplementary Information

Below is the link to the electronic supplementary material.

Supplementary Material 1

Supplementary Material 2

Supplementary Material 3

## Data Availability

All data generated or analyzed during this study are included in this published article [i.e., Supplementary File.xlsx, Data forward telemetry white cloth.xlsx, Data PV cell and Received power (White cloth).xlsx].
